# Combination of preoperative CA19-9 levels, cell differentiation, and age predicts survival for patients with gastric cancer before surgery

**DOI:** 10.1097/MD.0000000000028017

**Published:** 2021-12-10

**Authors:** Hui Hui Yin, Meng Qing Xu, Bin Zheng Liu, Lin Tao, Ya Jing Ma, Feng Li, Wen Jie Zhang

**Affiliations:** a Department of Pathology, the First Affiliated Hospital, Shihezi University School of Medicine, Shihezi, Xinjiang, China; b The Key Laboratories for Xinjiang Endemic and Ethnic Diseases, Shihezi University School of Medicine, Shihezi, Xinjiang, China; c Department of Pathology, Wuxi Branch of Ruijin Hospital Affiliated to Shanghai Jiao Tong University School of Medicine/Xinrui Hospital of Xinwu District, Wuxi, Jiangsu, China; d Department of Gastroenterology, Jinling Hospital, Nanjing, Jiangsu, China; e Department of Clinical Laboratories, the First Affiliated University Hospital, Shihezi University School of Medicine, Shihezi, Xinjiang, China; f Department of Pathology, Beijing Chaoyang Hospital, the Capital Medical University, Beijing, China.

**Keywords:** age, carbohydrate antigen 19-9, cell differentiation, gastric cancer, survival prognosis

## Abstract

Gastric cancer (GC) is very common in China, posing a threat to public health, with high morbidity and mortality ranks. Tumor-node-metastasis (TNM) staging system is routinely used to predict prognosis for patients with GC but only available after surgery. Therefore, searching for markers that can predict prognosis of GC patients before surgery is desirable to assist management decisions preoperatively. Among 322 GC patients followed-up for 128 months, the tumor markers alpha fetoprotein, carcinoembryonic antigen, carbohydrate antigen 19-9 (CA19-9), carbohydrate antigen 15-3 and carbohydrate antigen 72-4 of 168 patients were detected before surgery, and their impact on survival was analyzed. Four major findings were revealed: (1) Preoperative examined CA19-9 levels and cell differentiation using endoscopic biopsies were positively correlated with lymphatic metastases and TNM stages obtained after surgery. (2) Kaplan-Meier analyses demonstrated that poor survival of patients with GC was associated with higher CA19-9 levels, poor cell differentiation, and older age. (3) Cox multi-factorial regression analyses indicated that, in terms of predicting overall survival for GC patients, preoperative CA19-9 level, cell differentiation and age were independent factors, respectively, comparable to postoperative TNM staging system. (4) Using receiver operating characteristic curve analysis, we first revealed that preoperative CA19-9 levels and cell differentiation had the impact weights (IW) on survival comparable to postoperative TNM components. These findings suggest that preoperative CA19-9 levels, cell differentiation and age are useful prognostic related markers for GC patients, superior to postoperative TNM system in terms of timing for management. We propose that, assisted by clinical imaging, a comprehensive utilization of these preoperative survival-predictors may help formulate individualized medical management for GC patients such as surgical strategy, optimal chemotherapy and radiotherapy, and appropriate follow-up intervals after surgery.

## 1 Introduction

Gastric cancer (GC) is posing a threat to public health worldwide with higher incidence and mortality in less developed countries. GLOBOCAN 2018 estimates 456,124 new GC cases and 390,182deaths occurring in China, accounting for 44.1% and 49.9% of the world, respectively, and the morbidity and mortality rank second in malignant tumors9.^[1,2]^ Furthermore, there have been increased incidence and mortality of gastric cancer in China.^[3]^

Without routine screening, early diagnosis of GC is difficult and treatment of late-stage GC is often unsatisfactory, resulting in poor survival. Cancer invasion and metastasis are the major causes of death for GC patients and surgical removal of in situ tumors is a routine management. Tumor-node-metastasis (TNM) staging system, based on T, lymph node metastasis (N) and distant metastasis (M), is well established to predict prognosis for GC patients.^[4]^ However, pathological TNM staging (pTNM) cannot be obtained before surgery and the prognosis of patients with GC before surgery is evaluated difficultly. Although with lower accuracy, preoperatively defined clinical TNM (cTNM) may be correlated with prognosis of patients with GC. Therefore, Pre-assessment of prognosis for patients who underwent surgery, such as GC patients, has important clinical implications:

(1) Pre-assessing of prognosis for patients with GC serves as a warning sign that may assist formulation of surgical planning which in turn may improve the quality of surgery;
(2) Predicting prognosis may help design appropriate postoperative radio-chemotherapy^[5]^ and
(3) Determine optimal intervals of follow-up. It is desirable to identify markers that can predict poor prognosis before surgery.

There are several categories of markers that can be obtained before surgery, such as age,^[6]^ cell differentiation,^[7]^ tumor markers amongst others. Tumor markers, including specific proteins, glycoproteins, enzymes and hormones, originate from primary neoplasms and occasionally from tissues/organs affected by the cancer. Tumor markers are absent or present in small quantity in healthy tissues.^[8]^ A number of blood derived tumor markers are routinely tested before surgery for GC patients, such as carcinoembryonic antigen (CEA), carbohydrate antigen 19-9 (CA19-9), alpha fetoprotein (AFP), carbohydrate antigen 72-4 (CA72-4), and carbohydrate antigen 15-3 (CA 15-3). Serum levels of tumor markers play significant roles in cancer diagnosis, the evaluation of response to chemotherapy, the early detection of recurrence and metastasis.^[9–13]^ In GC patients, adverse prognosis has been suggested to be associated with poorly cell differentiation obtained via gastroscopic biopsy.

Our previous studies have revealed that preoperatively determined body mass index, blood albumin, triglycerides, bilirubin, and aminotransferase can predict survival for GC patients.^[14,15]^ Therefore, We have hypothesized that tumor markers obtained before surgery, individual or combined, may also be able to predict prognosis among GC patients. This study has tested the hypothesis and obtained several interesting findings:

(1) Levels of CA19–9 and degree of cell differentiation are positively correlated with the number of metastatic lymph nodes and TNM clinical stages.
(2) Poor survival of GC patients was associated with higher CA19-9 levels, poorly cell differentiation, and older age.
(3) In terms of predicting overall survival among GC patients, CA19-9 level, cell differentiation and age are independent factors, respectively, similar to postoperative determinants, such as M and TNM stages.
(4) Because CA19-9 levels, cell differentiation and age are obtained before surgery, these findings will not only provide advice for the doctor to choose a treatment strategy for the patient, but also assist in the formulation of individualized medical treatment after surgery.

## 2 Materials and methods

### 2.1 Patients and follow-up

GC patients (322) of ethnic Han nationality patients with histologically confirmed gastric cancer underwent surgery from 2004 to 2013 (last patient obtained in 2013) were collected from the First Affiliated Hospital of Shihezi University School of Medicine. The patients in this study were followed-up until 2016. All patients did not receive chemotherapy and/or radiotherapy before surgery. patients with GC After surgery were followed up for 122 months (12 years and 2 months) with a median follow-up time of 31 months. Among 322 GC patients, tumor markers were tested for 168 patients before surgery with complete clinical information (Table 1). Overall survival was defined from the date of surgery until the date of death or the date of the last follow-up. Patients who died within 30 days after surgery were defined as 0 month survival.

**Table 1:** Clinicopathological characteristics of age, cell differentiation, tumor markers, TNM stages, and follow-up information among 168 patients with gastric cancer.

	Patients Cohort (n = 168)			Patients Cohort (n = 168)	
Clinical Characteristics	n	%	Characteristics	n	%
Gender			CA72–4 (U/mL)		
Male	128	76.2	Range	0.1-600	
Female	40	23.8	Median	2.285	
Age, yr			Reference range	0-600	
Range	32–81		<6.9	131	79.9
Median	63		≥6.9	33	20.1
Mean ± SD	62.53 ± 10.92		CA15–3 (U/mL)		
Cell differentiation			Range	2.01–110.02	
Well	7	4.2	Median	7.295	
Moderately	50	29.8	Reference range	0-25	
Poorly	111	66.1	<25	158	97.5
CA19–9 (U/mL)			≥25	4	2.5
Range	0.3-2000		depth of Invasion		
Median	8.295		T1	20	11.9
Reference range	0-27		T2	31	18.5
<27	133	79.2	T3	110	65.5
≥27	35	20.8	T4	7	4.1
CEA (ng/mL)	2		Lymph node metastasis		
Range	0.1–247.1		Yes	63	37.5
Median	2.0		No	105	62.5
Reference range	0–5		Distant metastases		
<5	141	83.9	Yes	13	7.7
≥5	27	16.1	No	155	92.3
AFP (IU/mL)			TNM staging		
Range	0.25-53		I	38	22.6
Median	1.92		II	52	31.0
Reference range	0–5.6		III	64	38.1
<5.6	159	95.2	IV	14	8.3
≥5.6	8	4.8	Follow-up, months		
Survival outcome			Range	0-122	
alive	95	56.5	Median	31	
dead	73	43.5	Mean ± SD	32.94 ± 21.59	

### 2.2 Ethical standards

This study was approved by the Institutional Ethics Review Board (IERB No. SHZ2011LL10) at our First Affiliated Hospital, Shihezi University School of Medicine. Patients’ informed consents were obtained orally by phone during follow-up communications and standard university hospital guidelines in accordance with the Declaration of Helsinki including confidentiality and anonymity were followed in the handling and publication of patients’ tissues. IERB board agreed to obtain informed consent by phone.

### 2.3 Informed consent

Patients’ informed consent was obtained orally by phone during follow-up communications.

### 2.4 Classifications of gastric cancer

Gastric adenocarcinoma was classified according to the histopathological classification criteria of the World Health Organization (WHO, 2000) as follows: highly, moderately, and poorly differentiated adenocarcinomas.^[16]^ TNM (tumor-node-metastasis) clinical staging (I-IV), depth of invasion (T1-T4), lymphatic and distant metastases were defined according to the American Joint Committee on Cancer (the 8th edition, 2017)^[17]^ (Table 1).

### 2.5 Examination of tumor markers

Before surgery, heparinized blood was drawn from patients after fasting overnight, centrifuged and the serum was stored at -20°C until test. Tumor markers (CEA, CA19-9, AFP, CA72-4, carbohydrate antigen 15-3) were tested using electrochemical luminescence method on a Roche E170 Automatic Analyzer (Roche, Shanghai, China). According to the National Clinical Laboratory Procedures (the 3rd Edition),^[18]^ the cut-off values of normal reference ranges for the tumor markers tested before surgery were shown in Table 1.

### 2.6 Statistical analyses

Data analyses were performed using the statistical software package SPSS (version 20.0, IBM Corporation, Armonk, NY). The spearman ranking correlation method was employed to identify correlations among preoperative and/or postoperative variables. Chi-square (χ^2^) test and Fisher exact test were used to analyze differences between clinicopathological variables. Univariate and multivariate analyses of the relative prognostic importance of the parameters were performed using the Cox proportional hazards model. Kaplan-Meier method was used to calculate and plot survival curves, and a two-sided log-rank test was used to evaluate differences in survival curves. In addition, we introduced the receiver operating characteristic (ROC) curve and the area under the curve (AUC) to quantify the impact weights (or powers) of survival-predicting factors in terms of their differential abilities to predict survival. The larger the AUC, the more powerful the factor can be used to predict prognosis. All *P* values were 2-sided and differences with *P* < .05 were considered statistically significant.

## 3 Results

### 3.1 GC patients with advanced TNM stages show poor survival prognosis

**Figure 1:**
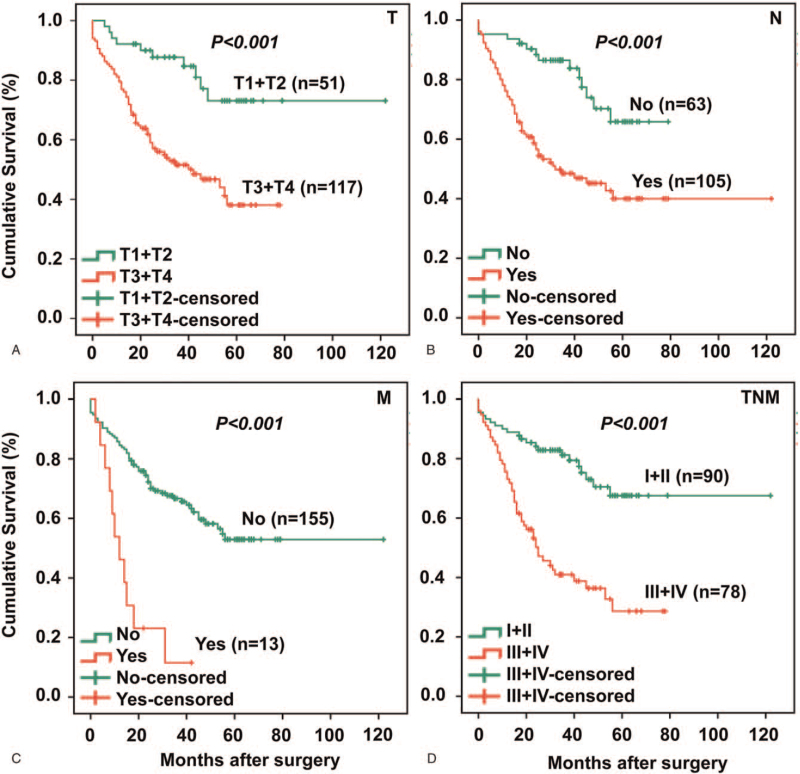
Conventional TNM risk factors that affect survival validate this patient cohort for new risk factors. The cohort included 168 GC patients, of whom 95 were alive and 73 were dead at the last follow-up. To validate the cohort for survival analysis, general survival is analyzed against well-established TNM staging system components that affect survival in GC patients using Kaplan-Meier method. Panel T shows the impact of varying degrees of cancer infiltration depth (T1, T2, T3, and T4) on the survival with T3 + T4 patients showing poorer survival than T1 + T2 patients. Panel N demonstrates that patients with lymph node metastasis have poorer survival than patients without the metastasis. Panel M indicates that patients with distant metastasis have worse survival than patients without distant metastasis. Finally, Panel TNM staging depicts a typical survival pattern that patients with early stages I + II have better survival than patients with advanced stages III + IV. T, N, M, and TNM staging were defined according to the AJCC TNM staging system. AJCC = American Joint Committee on Cancer.

This cohort included 168 GC patients with 95 alive and 73 dead by the last follow-up. To validate the reliability of clinical data in terms of prognosis of GC, we first analyzed the relationship between overall survival of these GC patients and several well-established conventional risk factors that are capable of predicting prognosis using Kaplan-Meier method and Log-rank test. As displayed in Figure 1, it was immediately clear that TNM staging system, including depth of invasion (T), N, M, and TNM stages, significantly impacted on overall survival of these patients with GC. The prognostic value of traditional risk factors that affect prognosis in this study has been verified, we carried on to analyze those less known risk factors or the combination of risk factors for survival as described below.

### 3.2 Preoperative age, cell differentiation, and CA19-9 levels impact on survival of GC patients

**Figure 2:**
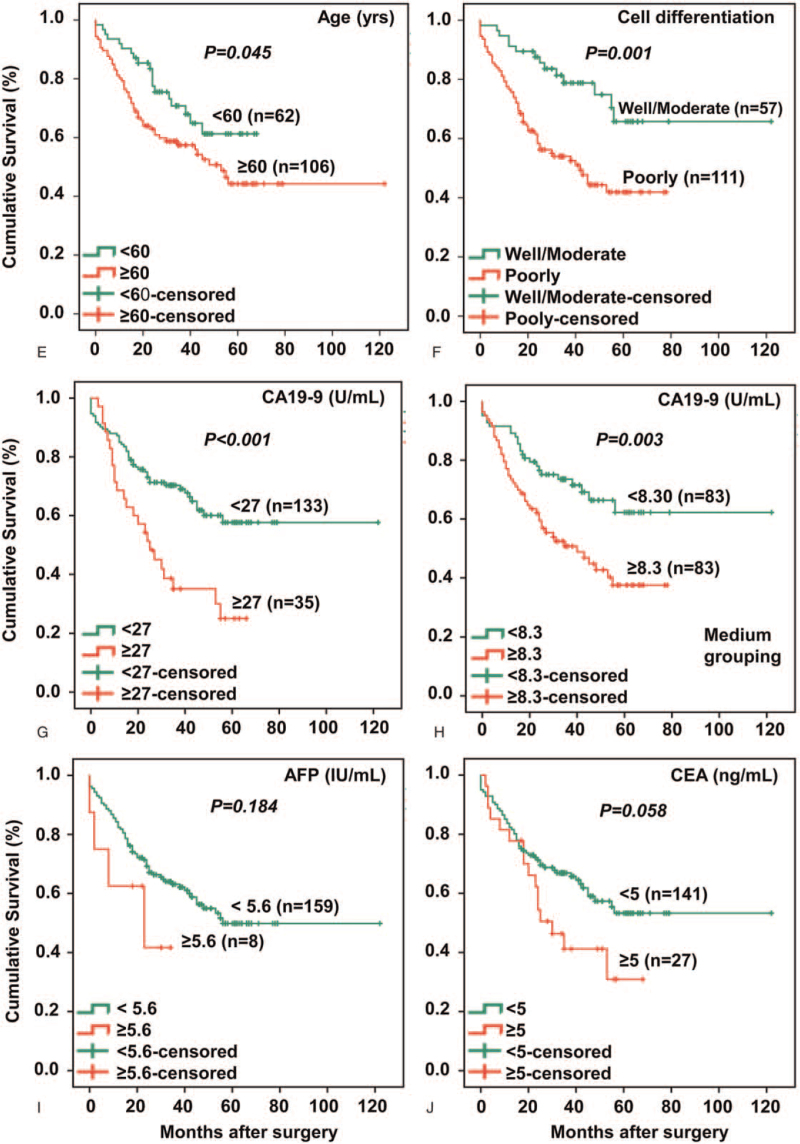
Older age, poor cell differentiation and higher levels of CA19-9 correlate with poor survival for GC patients. As shown in panel A, GC patients with an age of ≥ 60 years have a lower survival rate than GC patients with a younger age (<60 years). In panel B, GC patients with poor cell differentiation have a significantly poorer survival prognosis than those with well/moderate cell differentiation. In panel C (grouping by the reference range) and panel D (grouping by the median), it is obvious that GC patients having higher levels of CA19-9 show poorer survival than those having lower levels of CA19-9. These comparisons demonstrate that high levels of CA19-9, no matter defined by the reference range or defined by the median, can impact on survival of GC patients. Although there is a trend, blood levels of AFP (panel E) and CEA (panel F) do not significantly affect survival in this patient cohort. Blood levels of tumor markers CA12-5 and CA15-3 have no impact on survival of the GC patients analyzed (survival curves not shown). CA15-3 = carbohydrate antigen 15-3.

As shown in Figure 2, Kaplan-Meier survival curve indicted that age and cell differentiation significantly affected overall survival (Fig. 2A, B). Furthermore, poor survival of GC patients was associated with higher CA 19-9 levels (above clinical reference range, Fig. 2C). Similarly, when CA19-9 lenel was grouped by the median (cut-off value 8.3 U/mL), GC patients with higher levels of CA19-9 showed poorer survival than those with lower CA-19-9 levels (Fig. 2D). The above observations suggested that no matter by median grouping or by reference range grouping, the higher the CA19-9 level, the poorer the survival prognosis, that is, higher blood levels of CA19-9 would be a risk factor for survival of patients with GC. In addition, blood levels of tumor markers CEA (Fig. 2E) and AFP (Fig. 2F) were not significantly associated with survival outcomes among these GC patients.

### 3.3 Age, cell differentiation, and CA19-9 are independent factors affecting the survival of GC patients

As shown in Table 2, univariate analyses demonstrated that the prognosis and survival of patients with GC was associated with (i) clinical factors obtained before surgery including age (*P* = .03), cell differentiation (*P* < .001), CA19-9 levels (*P* = .001); (ii) conventional factors obtained after surgery including invasion depth (*P* < .001), N (*P* < .001), M (*P* < .001), and TNM staging (*P* < .001). However, the prognosis of GC patients was not associated with sex and blood levels of tumor markers CEA, AFP, CA72-4, carbohydrate antigen 15-3 (*P* >.05). Furthermore and as expected, multivariate analyses showed postoperative factors including M (hazard ratio [HR] = 2.46, *P* = .014) and advanced TNM stages (HR = 2.46, *P* = .001) were independent factors predicting survival of patients with GC. It was interesting to note that preoperative factors including age (HR = 2.10, *P* = .007), cell differentiation (HR = 2.84, *P* = .001), and CA19-9 (HR = 1.73, *P* = .049) also independently acted on survival for patients with GC.

**Table 2:** Similar to postoperative TNM staging, preoperative markers of age, cell differentiation and CA19–9 levels are independent predictors for survival prognosis in patients with GC as revealed by multivariate analyses.

	Univariate analysis			Multivariate analysis		
Clinico-pathological characteristics	HR	95% CI	*P* value	HR	(95% CI)	*P* value
Gender (Female vs Male)	1.09	(0.77, 1.54)	.62	/	/	/
Age (<60 vs ≥60 yrs)	1.43	(1.03, 1.98)	.03	2.10	(1.23, 3.61)	.007
Cell differentiation (Poorly vs Moderately/Well)	1.87	(1.32, 2.64)	<.001	2.84	(1.55, 5.21)	.001
CEA (<5 vs ≥5ng/mL)	1.70	(0.97, 2.97)	.06	/	/	/
CA19–9 (<27 vs ≥27U/mL)	2.34	(1.43, 3.85)	.001	1.73	(1.00, 3.00)	0.049
AFP (<5.6 vs≥5.6IU/mL)	1.96	(0.71, 5.41)	.20	/	/	/
CA72–4 (<6.9 vs ≥6.9U/mL)	1.00	(0.55, 1.79)	.98	/	/	/
CA15–3 (<25 vs ≥25U/mL)	0.61	(0.08, 4.40)	.62	/	/	/
Depth of invasion (T1/T2 vs T3/T4)	2.76	(1.87, 4.07)	<.001	/	/	/
Lymphatic metastasis (Yes vs No)	2.21	(1.59, 3.07)	<.001	/	/	/
Distant metastasis (Yes vs No)	3.59	(2.37, 5.43)	<.001	2.46	(1.20, 5.07)	.014
TNM staging (I/II vs III/IV)	3.08	(2.25, 4.22)	<.001	2.46	(1.42, 4.27)	.001

### 3.4 Correlations between CA19-9 levels and postoperative survival predictors

Having demonstrated a role of CA-19-9 in predicting the survival and prognosis of GC patients, we tested the hypothesis that CA-19-9 levels should be relevant to postoperative conventional survival predictors such as metastasis and TNM stage (as shown in Fig. 1). We found that there were 35 patients (19.6%) whose CA 19-9 levels were above the reference range (≥27 U/mL). Indeed as hypothesized, GC patients carrying lymphatic metastasis, M and late TNM stages exhibited higher levels (≥27 U/mL) of CA 19-9 (Table 3), which was shown to be an independent factor affecting survival of GC patients (Table 2, Fig. 2).

**Table 3:** Patients with lymphatic metastasis, distant metastasis and late TNM stages have higher levels (≥27 U/mL) of CA19-9.

	CA19–9			
	<27 U/mL	≥27 U/mL		
Clinicopathologic variable factors	n (%)	n (%)	*χ* ^2^	*P* value
Gender				
Male	33 (54.1)	28 (45.9)	0.35	.66
Female	100 (93.5)	7 (6.5)		
Age (yr)			0.57	.56
<60	51 (82.3)	11 (17.7)		
≥60	82 (77.4)	24 (22.6)		
Differentiation				
Well	7 (100.0)	0 (0.0)	3.65	.16
Moderately	36 (72.0)	14 (28.0)		
Poorly	90 (81.1)	21 (18.9)		
Depth of invasion				
T1	19 (95.0)	1 (5.0)	/	.17
T2	26 (83.9)	5 (16.1)		
T3	82 (74.5)	28 (25.5)		
T4	6 (85.7)	1 (14.3)		
Lymphatic metastasis				
No	57 (90.5)	6 (9.5)	7.82	.01
Yes	76 (72.4)	29 (27.6)		
Distant metastasis				
No	126 (81.3)	29 (18.7)	/	.03
Yes	7 (53.8)	6 (46.2)		
TNM staging				
I	35 (92.1)	3 (7.9)	10.97	.02
II	44 (84.6)	8 (15.4)		
III	46 (71.9)	18 (28.1)		
IV	8 (57.1)	6 (42.9)		

### 3.5 Correlations between preoperative and postoperative survival predictors

Age, cell differentiation and CA-19-9 were associated with the survival and prognosis of GC patients, we further hypothesized that the above preoperative survival predictors would show correlations with conventional postoperative ones, namely the TNM system. Not surprisingly, this hypothesis was validated by the following observations as shown in Table 4: (i) Positive correlations of CA-19-9 were found with N (r = 0.216, *P* < .001), M (r = 0.181, *P* < .05), and TNM staging (r = 0.252, *P* < .001). (ii) Positive correlations of preoperative cell differentiation were also observed with postoperative predictors including invasion depth (r = 0.223, *P* < .001), N (r = 0.242, *P* < .001), and TNM staging (r = 0.268, *P* < .001).

**Table 4:** Correlations of preoperative prognostic predictors of cell differentiation and CA 19–9 levels with postoperative TNM system.

		Preoperative predictors				Postoperative predictors			
	Variables (n = 168)	Gender	Age	Cell differentiation	CA19–9 levels	Depth of invasion	Lymph node metastasis	Distant metastasis	TNM staging
Preoperative predictors	Gender	1.000							
	Age	−0.134	1.000						
	Cell differentiation	0.013	−0.061	1.000					
	CA19–9 levels	−0.046	0.123	−0.047	1.000				
Postoperative predictors	Depth of invasion	−0.009	0.020	**0.223** ^∗∗^	0.136	1.000			
	Lymph node metastasis	−0.115	0.028	**0.242** ^∗∗^	**0.216** ^∗∗^	**0.386** ^∗∗^	1.000		
	Distant metastasis	0.100	0.003	0.056	**0.181** ^∗^	0.149	**0.178** ^∗^	1.000	
	TNM staging	−0.058	−0.041	**0.268** ^∗∗^	**0.252** ^∗∗^	**0.740** ^∗∗^	**0.719** ^∗∗^	**0.484** ^∗∗^	1.000

### 3.6 Quantify predicting powers for survival predictors by using ROC curves

**Figure 3:**
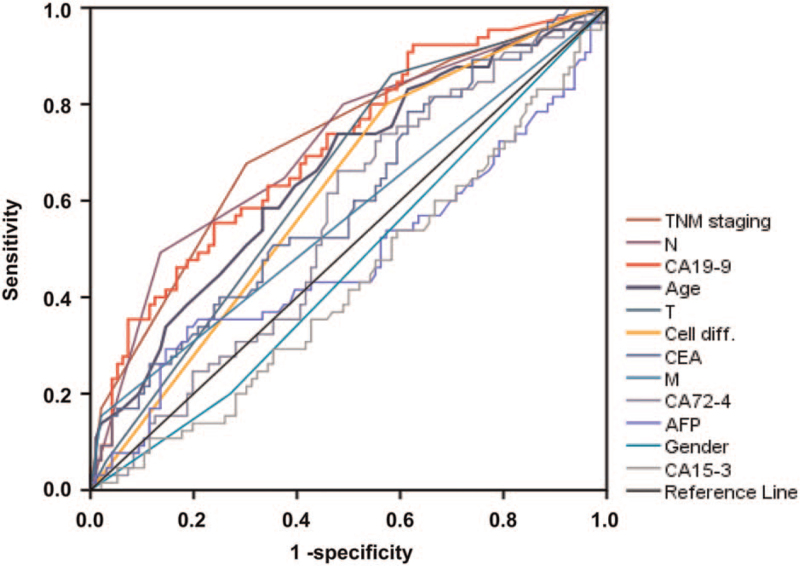
ROC curves reveal performance abilities of 8 risk factors affecting survival of GC patients. As shown, the diagonal black line in the middle is the reference line with an AUC of 0.500. TNM staging has the largest AUC (0.717, burgundy line), followed by N (0.712, purple line), CA19-9 (0.707, red line), age (0.655, blue line), T (0.642, light blue line), cell differentiation (0.617, orange line) and AUCs for the rest of factors are displayed in Table 5. It is very interesting to note that 3 non-conventional survival predictors, namely age, cell differentiation and CA19-9, have AUCs similar to, or even better than, AUCs of some conventional survival predictors of the TNM staging components (see Table 5). In keeping with the survival comparisons in Figures 1 and 2, these AUCs suggest that age, cell differentiation and CA19-9 have very similar, if not the same, power in predicting survival prognosis as do the TNM components. Cell diff. = cell differentiation, T= tumor invasion depth, N = lymph node metastasis, M = distant metastasis, TNM = tumor-node-metastasis.

As revealed in Figure 3, the large differences were showed in ROC curves defined by their sensitivity and specificity for 12 potential factors tested before surgery and their corresponding AUCs were displayed in Table 5. In this analysis, the volume of an AUC represents the weight (power) of a potential factor in terms of predicting survival prognosis, which we termed as impact weight (IW) (Table 5). IW is a function in terms of quantifying the relative power of a survival risk factor in a particular cancer. It was surprising to note that preoperatively determined CA-19-9 lever had a comparable AUC (0.707) to well-established TNM staging (0.717) and lymphatic metastasis (0.712), demonstrating their comparable impact weights in terms of predicting survival (Table 5). Furthermore, preoperatively determined factors, cell differentiation and age, also showed similar AUCs to those of TNM staging system although to a lesser degree. On the other hand, the M had a lower AUC (0.567) than those of invasion depth and N of the TNM system (Table 5), but uneven distributions of patients in Kaplan-Meier curves may be one, if not the only reason (Fig. 1C).

**Table 5:** Preoperatively determined CA19–9, age and cell differentiation have similar the AUCs or impact weights to postoperative TNM system in predicting survival prognosis of patients with GC.

Survival predictors	AUC (IW)	95% CI	*P* value
Reference curve	0.500	N/A	N/A
TNM staging	0.717	(0.636, 0.798)	**<.001**
Lymphatic metastasis	0.712	(0.630, 0.794)	**<.001**
CA19–9	0.707	(0.626, 0.788)	**<.001**
Age	0.655	(0.569, 0.741)	**.001**
Depth of invasion	0.642	(0.557, 0.728)	**.002**
Cell differentiation	0.617	(0.530, 0.703)	**.012**
CEA	0.598	(0.509, 0.687)	**.036**
Distant metastasis	0.567	(0.474, 0.659)	.153
CA72–4	0.555	(0.466, 0.644)	.238
AFP	0.480	(0.384, 0.575)	.663
Gender	0.465	(0.374, 0.555)	.446
CA15–3	0.428	(0.338, 0.518)	.121

## 4 Discussion

The survival time of GC patients after surgery is a critical barometer evaluating the effectiveness of diagnosis and management. The 5-year survival rates of gastric cancer in Japan and Korea had reached 60.3% and 68.9%, respectively. However, the 5-year survival rate in China is only 35.9%, with a significant gap with the developed countries in the world,^[19]^ Like all other cancers, postoperative survival of GC patients is a complex function determined by comprehensive interactions among multiple factors, known and unknown. Therefore, it is undoubtedly of great significance to carry out research on the factors affecting the prognosis of GC patients.^[20]^

Postoperative TNM staging system, based on T, N, and M is well-established to be reliable prognostic factors (4). In this study, we have first validated the GC patient cohort using TNM staging systemby survival analysis. As can be seen in Figure 1, poor cumulative survival rate of GC patients is correlated with invasive T3+T4, N, M, and advanced TNM staging III+IV, respectively, confirming previous observations.^[21]^ However, TNM staging is only a index that can only be confirmed after surgery, which can only provide a theoretical basis for postoperative treatment strategies, and it is of little significance for individual patients to plan surgical strategies before surgery. Therefore, it is desirable to look for bio-markers before surgery that (i) are routinely tested; (ii) have relationships with TNM staging system; and (iii) are able to predict the survival and prognosis of GC patients. Based on these criteria, five preoperative tumor markers were detected, such as CA19-9, CEA, CA12-5. We analyzed their relationships with conventional TNM staging as well as their abilities to predict the survival and prognosis of patients with GC before surgery.

We have hypothesized that, if preoperative tumor markers and cell differentiation have impact on the survival of patients with GC, they should be correlated with one or more postoperative predictors which are well established to predict survival. Indeed and as expected, we have observed that positive correlations are shown between cell differentiation and T, N, and TNM staging, respectively, and CA19-9 levels are positively correlated with N, M, and TNM staging, respectively. They may suggest there are intrinsic relationships between TNM system and CA19-9 levels and cell differentiation. In other words, the status of CA19-9 and cell differentiation may imply, before surgery, ongoing cancer progression or the potential of cancer progression prior to TNM staging.

These above correlations, on the other hand, further suggest that cell differentiation and CA19-9 levels may be able to predict prognosis of patients with GC just as TNM system (Fig. 1). Indeed as shown in Figure 2, patients with ≥ 60 years of age appear to show inferior survival compared with patients with < 60 years. Patients with poorly cell differentiation, which is diagnosed by gastroscopic biopsy before surgery, show poor survival. Higher blood levels of tumor marker CA19-9, either grouped by the clinical reference range or by the median, render GC patients poorer survival than those with lower levels of CA-19-9 (Fig. 2C, D), in keeping with the correlation analyses described above and with previous studies.^[22]^ Our observations shown CA-19-9 can be used to as a well prognostic marker for GC patients. In addition, the higher the CEA level, the lower the survival rate, but the observation is not statistically significant (Fig. 2F). Tumor marker AFP (Fig. 2E) has failed to show impact on survival of GC patients which is in line with previous findings.^[12]^

In our cohort, 19.6% of GC patients carry elevated levels of CA19-9, which is in the lower end as compared with other GC patient populations (16.0% to 34.6%).^[23,24]^ In general as seen in Table 3, GC Patients with lymphatic metastasis, M, and advanced TNM stages have higher blood levels (≥27 U/mL) of CA19-9. For example, higher levels of CA19-9 are more frequently seen in GC patients diagnosed as having higher TNM stages III and IV (68.5%) compared with GC patients diagnosed as having lower TNM stages I and II (31.5%). Furthermore, Cox proportional hazards regression analysis indicates that GC patients with elevated levels of CA19-9 are at a risk of death 1.73 times higher than GC patients with normal reference levels of CA19-9, which appears to support the survival impact of CA19-9 on GC patients (Fig. 2). These observations corroborates some previous findings.^[25,26]^

As shown in Table 2, it is important to note that multivariate analyses indicate 3 preoperative factors, namely age, cell differentiation, and CA 19-9 to behave as independent prognostic predictors, comparable to postoperative factors of M and TNM staging, further strengthening the case of those survival observations as shown in Figure 2. To compare discriminative powers between or among different categories of risk factors so as to score the differences in their powers of predicting survival prognosis, we have first introduced ROC curve and AUC to quantify (score) the power of a survival predictor in gastric cancer (Fig. 3). As shown in Table 5, It is important to note that, preoperatively obtained marker CA19-9 carries an IW (0.707) to those of well-established TNM staging (0.717) and lymphatic metastasis (0.712), demonstrating CA19-9 to be a comparable factor to TNM staging components in terms of survival prediction. Furthermore, the other two factors obtained before surgery, cell differentiation and age, also carry similar IWs/AUCs to those of the TNM components although to a lesser degree.

In summary, our study demonstrates that, in gastric cancer, preoperative CA19-9 levels and cancer cell differentiation are positively correlated with the well-established TNM components. Furthermore, it should be emphasized that cancer cell differentiation and, especially CA19-9, have comparable prognostic powers to the components of the golden standard TNM staging system by using ROC/AUC analysis. Accordingly, we introduce the concept of impact weight (IW) in terms of prognosis, which may be useful to compare across a spectrum of individual survival risk factors in a particular cancer that can benefit the clinical selection of the best prognostic factors for cancer patients of interest.

Predicting prognosis for cancer patients before surgery has clinical implications. Because information of CA19-9 levels, cell differentiation, and age is available before surgery, they may serve as useful “pre-warning indicators” in clinical management decisions before surgery. For example, when combined with other examinations such as clinical imaging, these “pre-warning indicators” obtained before surgery can be administered to patients with individualized surgical planning, the best radiotherapy and chemotherapy dose and time, and appropriate follow-up interval can be determined. Although it varies from patient to patient, chemo-radiotherapy has serious side-effects which often affect patients’ quality of life and perhaps prognosis of patients. Therefore, if a patient is suggestive of having a poor prognosis by preoperative markers or marker combinations, it may implicate to consider appropriate dosage and course length of chemo-radiotherapy which may differ from routine guidelines. This hypothesis surely warrants further clinical studies in patients with GC. On the other hand, for those patients who are suitable for surgery, detecting these prognostic predictors may have implications in patients’ palliative care program.

There exist several limitations in this study. Besides markers examined, there are also other recently proposed candidate substances such as cytokines (IL-6, IL-8, IL-17) that are indicative to have high clinical diagnosis and prognosis value as biomarkers of gastric cancer.^[27–30]^ With the increase in the typys of detected markers, the establishment of a combined detection and prediction model for patients with gastric cancer will be very necessary in the future. In addition, limited sample size and a single-center design may reduce the power of statistical analysis and the accuracy of results. and as possible confounding factors, such as smoking and infections, may affect some tumor markers, future studies should consider collecting information of possible confounding factors for similar analysis. Furthermore, surgery-related factors, such as the quality of lymphadenectomy, surgical radicalness, extent of gastrectomy and associated resections, may affect prognosis in patients with GC, which should be investigated whenever possible in future studies.

## Acknowledgments

The authors express thanks to Yan Wang and Yu Ma for their help in patient follow-ups.

## Author contributions

**Conceptualization:** Hui Hui Yin, Meng Qing Xu, Wen Jie Zhang.

**Data curation:** Hui Hui Yin, Meng Qing Xu, Bin Zheng Liu, Lin Tao.

**Formal analysis:** Hui Hui Yin, Meng Qing Xu, Bin Zheng Liu.

**Funding acquisition:** Wen Jie Zhang.

**Investigation:** Hui Hui Yin, Meng Qing Xu.

**Methodology:** Hui Hui Yin, Meng Qing Xu, Ya Jing Ma, Feng Li, Wen Jie Zhang.

**Project administration:** Ya Jing Ma, Wen Jie Zhang.

**Resources:** Lin Tao, Ya Jing Ma, Feng Li, Wen Jie Zhang.

**Software:** Hui Hui Yin, Meng Qing Xu, Bin Zheng Liu.

**Supervision:** Bin Zheng Liu, Wen Jie Zhang.

**Validation:** Hui Hui Yin, Meng Qing Xu, Wen Jie Zhang.

**Visualization:** Hui Hui Yin, Meng Qing Xu, Wen Jie Zhang.

**Writing – original draft:** Hui Hui Yin, Meng Qing Xu, Lin Tao, Wen Jie Zhang.

**Writing – review & editing:** Hui Hui Yin, Meng Qing Xu, Feng Li, Wen Jie Zhang.
